# Theoretical and Experimental Investigation of 3D-Printed Polylactide Laminate Composites’ Mechanical Properties

**DOI:** 10.3390/ma16227229

**Published:** 2023-11-19

**Authors:** Arthur E. Krupnin, Arthur R. Zakirov, Nikita G. Sedush, Mark M. Alexanyan, Alexander G. Aganesov, Sergei N. Chvalun

**Affiliations:** 1 National Research Centre “Kurchatov Institute”, 123182 Moscow, Russia; nsedush@gmail.com (N.G.S.);; 2 Department of Applied Mechanics (RK-5), Faculty of Robotics and Complex Automation, Bauman Moscow State Technical University, 105005 Moscow, Russia; 3 Petrovsky National Research Centre of Surgery, 119435 Moscow, Russia

**Keywords:** additive manufacturing, Tsai–Hill failure criterion, classical laminate theory, digital image correlation, polylactide, laminate composites

## Abstract

The purpose of this work is to theoretically and experimentally investigate the applicability of the Tsai–Hill failure criterion and classical laminate theory for predicting the strength and stiffness of 3D-printed polylactide laminate composites with various raster angles in mechanical tests for uniaxial tension and compression. According to the results of tensile and compression tests, the stiffness matrix components of the orthotropic individual lamina and strength were determined. The Poisson’s ratio was determined using the digital image correlation method. It was found that the Tsai–Hill criterion is applicable for predicting the tensile strength and yield strength of laminate polymer composite materials manufactured via fused deposition modeling 3D printing. The calculated values of the elastic moduli for specimens with various raster angles correlate well with the values obtained experimentally. In tensile tests, the error for the laminate with a constant raster angle was 3.3%, for a composite laminate it was 4.4, in compression tests it was 11.9% and 9%, respectively.

## 1. Introduction

Polylactic acid (PLA) is a biodegradable and biocompatible polyester [1] that is widely used in medicine for bone scaffolds [2], stents [3], bone plates [4], and lumbar cage [5] manufacturing. The preparation of PLA-based composites with glass fibers [6], carbon fibers [7], and calcium phosphates [8] not only allows for the mechanical properties of the final products to be significantly improved but also facilitates the process of tissue regeneration [1]. Additive manufacturing significantly expands the application areas of PLA and composites. One of the most accessible and widespread technologies is extrusion-based fused deposition modeling (FDM) or fused filament fabrication (FFF) (as an alternative descriptor) 3D printing, which allows parts with a complex topology to be fabricated. Zhang Boqing et al. [9] used FDM 3D printing for manufacturing scaffolds for bone regeneration made of PLA/nano-hydroxyapatite (nHA). The 50% nHA composite displayed reliable printability and high printing accuracy. Its compressive strength exceeded that of human cancellous bone. Increasing the nHA content alleviated the acidity of PLA degradation products, modified the rate of degradation, and improved bioactivity. The composite scaffolds with high nHA loading demonstrated superior osteo-regeneration capabilities in vivo. Jiang et al. [10] fabricated PLA/316L composite scaffolds incorporating stainless steel particles ranging from 5 vol% to 15 vol%. The FFF process was utilized for this purpose. Implants and scaffolds made of composites based on PLA and polycaprolactone (PCL), which is another synthetic polymer widely used in medicine, are also promising. Compared to PCL, glassy PLA is inherently more fragile and biodegradable. Additionally, it exhibits lower elasticity and flexibility. Therefore, blending these two polymers can be a viable approach towards the creation of a novel biomaterial that can address the shortcomings of each individual polymer [11]. Elin Åkerlund et al. [12] investigated the use of a blend of PLA, PCL, and HA to create customized biocompatible and bioresorbable polymer-based composite filaments. The filaments were produced through extrusion and intended for use in FFF 3D printing. The findings demonstrated that the extrusion and printing process had no effect on the materials’ chemical composition. All materials displayed superior mechanical properties to those of human trabecular bone, even after undergoing degradation, with a mass loss of approximately 30% for polymer blends and 60% for composites. Liu et al. [13] fabricated nanocomposites of PLA/PCL that exhibit favorable thermo-responsive cyclic shape memory effects as well as favorable crystallization and mechanical properties for 3D printing. Zhang et al. [14] investigated the degradation behavior and mechanical properties of cellulose nanofiber (CNF)/PLA composites. The composites showed an 18.4% higher tensile modulus compared to neat PLA but a 7.4% lower strength as a result of poor interfacial bonding between the CNF and PLA. Additionally, the degradation process induced marked deformation in the samples due to residual stress created during the 3D printing procedure.

The manufacturing of medical devices places high demands on the strength and stiffness of implants and scaffolds made via FDM/FFF 3D printing, so the problem of their design and calculation is extremely relevant. Monaldo et al. [15] investigated the mechanical behavior of PLA samples printed via material extrusion. Different filament printing orientations and varying flow rate percentages are examined to determine their impact on mechanical performance. A new two-level numerical model for multiscale analyses is introduced. The macroscopic structural behavior of the 3D-printed component is explained via a laminate finite element model that employs the first-order shear deformation theory. The numerical results obtained are compared with the experimental results, showing the effectiveness of the proposed modelling approach. Stojković et al. [16] examined how different annealing conditions—time, temperature, and layer height—affect the tensile strength and dimensional alteration of three 3D-printed materials (namely, PLA, PETG, and reinforced PETG with carbon fiber). Samples with different layer heights (0.1 mm, 0.2 mm, and 0.3 mm) underwent annealing at temperatures ranging from 60 to 100 °C for 30, 60 and 90 min. Tensile tests were then conducted, and regression models were formulated to scrutinize the impact of these parameters on tensile strength. The results indicate that layer height has a greater impact on tensile strength compared to annealing time and temperature. Bartosiak et al. [17] conducted a comprehensive investigation into how filament orientation impacts the tensile stiffness of 3D-printed structures. Their study focused on 3D-printed structures made from PLA using FFF technology and subjected them to rigorous testing with a universal tensile testing machine Shimadzu AG-X plus (Duisburg, Germany). This approach combines representative volume element and classical laminate theory (CLT) techniques to extrapolate the mechanical properties of the test material. A comparative study of the results derived from analytical methods and experimental trials involving five series of samples with varied layups reveals that the newly proposed numerical method displays a stronger correlation with the experimental outcomes. It delivers a relative error margin of up to 8%. Torre et al. [18] examined the performance of short-length PLA 3D-printed structures under compression, with a particular emphasis on buckling. They carried out a comprehensive experimental study on square polymeric columns manufactured via FFF, with the longitudinal axis located in the out-of-plane direction in the 3D printing coordinate system. The critical slenderness ratio at which the structures start to buckle was determined. The experimental findings are compared to three analytical models for predicting buckling in isotropic materials, along with linear and non-linear finite element models. Across a wide range of slenderness ratios, the tangent modulus theory provides a reliable and cautious approximation of critical loads when used with compressive mechanical properties. Additionally, the non-linear FE models’ predictions are in line with the experimental results. Kiendl et al. [19] studied how the raster layup affects the properties of 3D-printed PLA materials produced through FDM. They showed that when using standard layups with alternating layer orientations of 90°, the stiffness and strength are almost isotropic. However, toughness showed a strong anisotropy. Additionally, the study found that the material’s behavior changes between brittle and ductile depending on the direction of the applied load. A novel scheme for stacking layers is proposed, which offers enhanced toughness and strength in comparison to standard approaches. Balderrama-Armendariz et al. [20] studied the torsional properties and shear strength of ABS-M30 specimens to observe the impact of additive manufacturing. The results showed that there was an interaction of factors in all the measured mechanical variables whenever an orientation and a raster angle were applied. In comparison to injection molding, the FDM specimens showed similarity in all measured torsion variables, excluding the fracture strain. Therefore, it can be concluded that the FDM process is capable of manufacturing components with similar elastic properties but reduced ductility when compared to injection molding. The orthotropic nature of FDM/FFF-printed parts has been established in works by Mishra et al. [21], Somireddy et al. [22], and Alaimo et al. [23]. Approaches based on the representation of 3D-printed parts as transversally isotropic are presented in the works by Yao et al. [24,25]. To predict the failure of FDM/FFF-printed parts, the Tsai–Hill criterion can be used [25,26]. The Tsai–Hill theory is considered an extension of the Von Mises failure criterion. The failure strengths in the principal material directions are assumed to be known [27]. Zhao et al. [28] put forth two innovative theoretical models for predicting the tensile strength and Young’s modulus of a PLA material manufactured through FDM additive printing, with variations in printing angles and layer thicknesses. Firstly, a strength theoretical model was established based on a transversely isotropic material hypothesis and the Tsai–Hill strength criterion. Secondly, a Young’s modulus theoretical model was established based on a orthotropic material hypothesis in the plane stress state. Test results have shown that the tensile strength and Young’s modulus of the FDM-additive-manufactured PLA material exhibit an increase with an increase in printing angle or a decrease in layer thickness. Fuchs et al. [29] conducted tests to determine the validity of the Tsai–Hill equation in microfibril-reinforced composites (MFCs) with diameters of approximately 1 μm and aspect ratios of around 100. The tensile tests were performed on cut specimens at various angles relative to the uniaxially aligned microfibrils to assess the level of agreement with Tsai–Hill predictions. The measured values are slightly higher than the calculated ones. This can be attributed to the higher aspect ratios of microfibrils, their more homogeneous distribution, and most importantly, the better matrix/reinforcement adhesion observed in MFCs when compared to common composites. The fracture mechanism, determined from scanning electron microscopy observations on the fracture surfaces, is also discussed, and a good agreement with the mechanical behavior is observed. Jin et al. [30] studied the room-temperature compressive strength and deformation mechanisms in a directional solidified Nb-Si alloy with different orientations. The Tsai–Hill criterion was applied to explain the anisotropy of the compressive strength of the alloy at an angle to the direction of the heat flow. Yang [31] investigated the Tsai–Hill strength criterion for unidirectional S-glass-, E-glass-, and graphite-fiber-reinforced composite plate specimens subjected to off-axis tension and compression. Good agreement with the experimental results was achieved, except for the case of off-axis shear, where large deviations occurred between the analytical and experimental results. Gawryluk et al. [32] examined the behavior of short, thin-walled carbon–epoxy angle columns subjected to uniform shortening. Both experiments and numerical analyses were carried out, covering the loading range until structural damage began. The numerical analyses involved several failure criteria, such as the Tsai–Hill criterion. The results showed that the Tsai–Hill criterion led to an underestimate of the damage initiation load.

Despite this, there is a lack of research investigating the mechanical behavior of laminate composites based on the CLT under both tension and compression in a comparative way. In this case, the anisotropy of the mechanical properties of FDM/FFF-printed composites may manifest itself more strongly [2]. It is therefore important to have reliable tools, methods, and algorithms to determine the mechanical properties of such materials. These studies are the starting point for the calculation of structures under complex loading conditions, including bending, buckling, etc. The utilization of PLA as a model material for solving such a problem is justified due to its availability, the reproducibility of its mechanical properties, and the convenience of 3D printing test samples. In this work, we theoretically and experimentally investigated the applicability of the Tsai–Hill failure criterion and CLT for predicting the strength and stiffness of 3D-printed PLA laminate composites with various raster angles in mechanical tests for uniaxial tension and compression. It was found that the Tsai–Hill criterion is applicable for predicting the ultimate tensile strength and compressive yield stress of laminate polymer composite materials manufactured via FDM/FFF 3D printing. Two different methods were used to determine the shear strengths and Poisson’s ratios based on the symmetry of the stiffness matrix. The novelty of the research also lies in the application of the CLT for the estimation of the Young’s modulus of laminate composites with different raster angles, both in terms of tension and compression. Finally, the results of the theoretical and experimental evaluations of the ultimate tensile strength, compressive yield stress, and Young’s moduli will be presented and thoroughly compared with the results of the full-scale experiments.

## 2. Materials and Methods

### 2.1. Theoretical Modeling of 3D-Printed Laminate Composites

In the general case of an anisotropic body, Hooke’s law can be written in the following form ([27]):

(1) $$\left\{\begin{array}{c} \varepsilon_{1}\\ \varepsilon_{2}\\ \varepsilon_{3}\\ \gamma_{23}\\ \gamma_{31}\\ \gamma_{12} \end{array}\right\}=\left[\begin{array}{cccccc} S_{11}\; & S_{12} & S_{13} & S_{14} & S_{15} & S_{16}\\ S_{21} & S_{22} & S_{23} & S_{24} & S_{25} & S_{26}\\ S_{31} & S_{32} & S_{33} & S_{34} & S_{35} & S_{36}\\ S_{41} & S_{42} & S_{43} & S_{44} & S_{45} & S_{46}\\ S_{51} & S_{52} & S_{53} & S_{54} & S_{55} & S_{56}\\ S_{61} & S_{62} & S_{63} & S_{64} & S_{65} & S_{66} \end{array}\right]\left\{\begin{array}{c} \sigma_{1}\\ \sigma_{2}\\ \sigma_{3}\\ \tau_{23}\\ \tau_{31}\\ \tau_{12} \end{array}\right\}$$

This 6 × 6 matrix [*S*] is a symmetric compliance matrix. For orthotropic materials, if the coordinate planes of the Cartesian coordinate system are combined with the planes of elastic symmetry, then the matrix will take the following form:

(2) $$[S]=\left[\begin{array}{cccccc} S_{11} & S_{12} & S_{13} & 0 & 0 & 0\\ S_{21} & S_{22} & S_{23} & 0 & 0 & 0\\ S_{31} & S_{32} & S_{33} & 0 & 0 & 0\\ 0 & 0 & 0 & S_{44} & 0 & 0\\ 0 & 0 & 0 & 0 & S_{55} & 0\\ 0 & 0 & 0 & 0 & 0 & S_{66} \end{array}\right]$$

This matrix can be represented by the elastic constants of the material (the Young’s moduli and Poisson’s ratios):

(3) $$[S]=\left[\begin{array}{cccccc} \frac{1}{E_{1}} & -\frac{\nu_{21}}{E_{2}} & -\frac{\nu_{31}}{E_{3}} & 0 & 0 & 0\\ -\frac{\nu_{12}}{E_{1}} & \frac{1}{E_{2}} & -\frac{\nu_{32}}{E_{3}} & 0 & 0 & 0\\ -\frac{\nu_{13}}{E_{1}} & -\frac{\nu_{23}}{E_{2}} & \frac{1}{E_{3}} & 0 & 0 & 0\\ 0 & 0 & 0 & \frac{1}{G_{23}} & 0 & 0\\ 0 & 0 & 0 & 0 & \frac{1}{G_{31}} & 0\\ 0 & 0 & 0 & 0 & 0 & \frac{1}{G_{12}} \end{array}\right]$$

Hooke’s law (1) for an orthotropic body in a plane stress state can be simplified to the following expression:

(4) $$\left\{\begin{array}{c} \varepsilon_{1}\\ \varepsilon_{2}\\ \gamma_{12} \end{array}\right\}=\left[\begin{array}{ccc} S_{11} & S_{12} & 0\\ S_{21} & S_{22} & 0\\ 0 & 0 & S_{66} \end{array}\right]\left\{\begin{array}{c} \sigma_{1}\\ \sigma_{2}\\ \tau_{12} \end{array}\right\}.$$

And the stresses from (4) can be expressed as follows:

(5) $$\left\{\begin{array}{c} \sigma_{1}\\ \sigma_{2}\\ \sigma_{12} \end{array}\right\}=\left[\begin{array}{ccc} E_{11} & E_{12} & 0\\ E_{21} & E_{22} & 0\\ 0 & 0 & E_{66} \end{array}\right]\left\{\begin{array}{c} \varepsilon_{1}\\ \varepsilon_{2}\\ \gamma_{12} \end{array}\right\}$$

The coefficients of the stiffness matrix in expression (5) can be rewritten using the elastic constants of the material in the next form:

(6) $$\begin{array}{c} E_{11}=\frac{E_{1}^{2}}{E_{1}-\nu_{12}^{2}E_{2}};E_{12}=\frac{\nu_{12}E_{1}E_{2}}{E_{1}-\nu_{12}^{2}E_{2}};\\ E_{22}=\frac{E_{1}E_{2}}{E_{1}-\nu_{12}^{2}E_{2}};E_{66}=G_{12}. \end{array}$$

**Figure 1:**
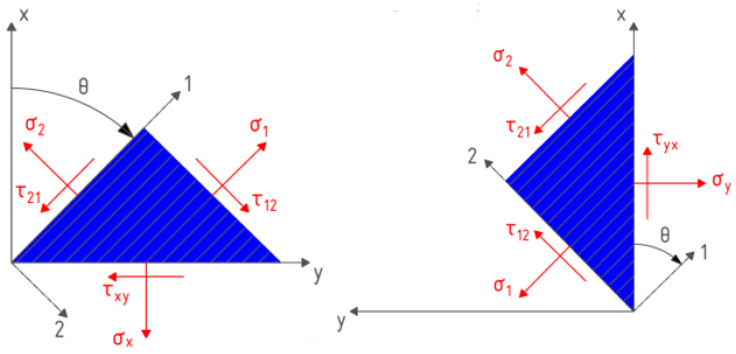
Rotation of the coordinate system by an angle θ.

Thus, the number of independent elastic constants necessary for the complete determination of the stiffness matrix for an orthotropic material in a plane stress state is reduced to four. When rotating the coordinate system by an angle *θ* (Figure 1) in the case of a plane stress state, the components of the stiffness matrix for an individual lamina can be derived as follows:

(7) $$\begin{array}{l} E_{11}^{*}=c^{4}E_{11}+s^{4}E_{22}+2(E_{12}+2E_{66})s^{2}c^{2};\\ E_{12}^{*}=(E_{11}+E_{22}-4E_{66})s^{2}c^{2}+(s^{4}+c^{4})E_{12};\\ E_{13}^{*}=[c^{2}E_{11}-s^{2}E_{22}+(E_{12}+2E_{66})(s^{2}-c^{2})]sc;\\ E_{22}^{*}=s^{4}E_{11}+c^{4}E_{22}+2(E_{12}+2E_{66})s^{2}c^{2};\\ E_{23}^{*}=[s^{2}E_{11}-c^{2}E_{22}-(E_{12}+2E_{66})(s^{2}-c^{2})]sc;\\ E_{33}^{*}=[E_{11}-2E_{12}+E_{22}]s^{2}c^{2}+(s^{2}-c^{2})E_{66}. \end{array}$$

where *c* = cos*θ* and *s* = sin*θ*.

According to the CLT [27,33], the following law holds for each *k*-th lamina:

(8) $${\left\{\sigma_{xy}\right\}}^{k}={\left[E^{*}\right]}^{k}{\left\{\varepsilon_{xy}\right\}}^{k}$$

The normal and shear forces arising in the cross section of a laminate composite with thickness t and coordinates of a *k*-th lamina *z_k_* can be determined as follows:

(9) $$\begin{array}{l} \left\{\begin{array}{c} N_{x}\\ N_{y}\\ N_{xy} \end{array}\right\}=\int_{-t/2}^{t/2}\left\{\begin{array}{c} \sigma_{x}\\ \sigma_{y}\\ \tau_{xy} \end{array}\right\}dz=\sum_{k=1}^{N}\int_{z_{k-1}}^{z_{k}}{\left\{\begin{array}{c} \sigma_{x}\\ \sigma_{y}\\ \tau_{xy} \end{array}\right\}}^{k}dz\\ =\sum_{k=1}^{N}\left(z_{k}-z_{k-1}\right){\left[E^{*}\right]}^{k}\left\{\begin{array}{c} \varepsilon_{x}^{0}\\ \varepsilon_{y}^{0}\\ \gamma_{xy}^{0} \end{array}\right\}+\frac{1}{2}\sum_{k=1}^{N}\left(z_{k}^{2}-z_{k-1}^{2}\right){\left[E^{*}\right]}^{k}\left\{\begin{array}{c} \varkappa_{x}^{0}\\ \varkappa_{y}^{0}\\ \varkappa_{xy}^{0} \end{array}\right\} \end{array}$$

Here [*ε*^0^] and [*æ*^0^] are the strains and curvatures for *z* = 0, respectively. And in the case of uniaxial tension or compression, it can be simplified:

(10) $$\left\{\begin{array}{c} N_{x}\\ 0\\ 0 \end{array}\right\}=[F]\left\{\begin{array}{c} \varepsilon_{x}^{0}\\ \varepsilon_{y}^{0}\\ \gamma_{xy}^{0} \end{array}\right\}$$

where

(11) $$F_{ij}=\sum_{k=1}^{N}E_{ij}^{{*}^{k}}(\theta )(z_{k}-z_{k-1})$$

After the inverse transformation, the strains can be expressed in the form:

(12) $$\left\{\begin{array}{c} \varepsilon_{x}^{0}\\ \varepsilon_{y}^{0}\\ \gamma_{x}^{0} \end{array}\right\}={\left[F\right]}^{-1}\left\{\begin{array}{c} N_{x}\\ 0\\ 0 \end{array}\right\}=[A]\left\{\begin{array}{c} N_{x}\\ 0\\ 0 \end{array}\right\}$$

Finally, the Young’s modulus of a composite with an orientation of laminas symmetrical with respect to the median plane can be written as follows:

(13) $$E_{x}=\frac{1}{A_{11}h}$$

### 2.2. Tsai–Hill Failure Criterion

In general, the Tsai–Hill criterion is formulated in the following form [24]:

(14) $$\frac{\sigma_{1}^{2}}{X^{2}}-\frac{\sigma_{1}\sigma_{2}}{X^{2}}+\frac{\sigma_{2}^{2}}{Y^{2}}+\frac{\tau_{12}^{2}}{W^{2}}=1$$

where *X* and *Y* are ultimate tensile strengths in the principal directions (or compressive yield stresses) and W is the shear strength. In the case of uniaxial loading, the maximum stress value, *σ_x_^max^*, as a function of the raster angle, *θ*, can be expressed as follows:

(15) $$\sigma_{x}^{max}(\theta )={\left[\frac{\cos^{4}\theta }{X^{2}}+\left(\frac{1}{W^{2}}-\frac{1}{X^{2}}\right)\sin^{2}\theta \cos^{2}\theta +\frac{\sin^{4}\theta }{Y^{2}}\right]}^{-\frac{1}{2}}$$

### 2.3. 3D Printing of Specimens with Various Raster Angles

Specimens for uniaxial tension (ISO 527-2 [34]) and compression (ASTM D-695 [35]) were manufactured with a Raise3D PRO2 3D printer. The slicing of the models was carried out via ideaMaker 4.0.1 software. A commercial poly-L-lactide REC (natural color) 1.75 mm filament (Moscow, Russia) was used as a material for manufacturing. The 3D printing parameters are highlighted in Table 1.

**Figure 2:**
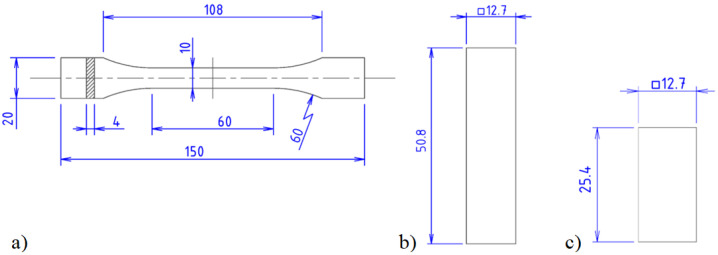
(**a**) Specimen for uniaxial tension, (**b**) specimen for Young’s modulus determination in uniaxial compression and (**c**) specimen for yield stress determination in uniaxial compression.

The sketches of the specimens are shown in Figure 2.

In the tensile tests, a Type 1B (Figure 2a) sample with a width of 10 mm was selected in order to fully take into account the influence of the raster angle orientation, while 2 types of samples were manufactured for compression tests: to determine the Young’s modulus (Figure 2b) and yield stress (Figure 2c). For the uniaxial tension and compression tests, the samples were 3D printed with raster angles *θ* ranging from 0° to 90° and a constant step of 15°, where 0° coincides with the direction of the applied load, x (Figure 1). To assess the applicability of the CLT, laminates with symmetrical lamina orientation were also manufactured: [0°, 90°, …] and [0°, 45°, 60°, 90°, 0°, −45°, −60°, −90°, …].

### 2.4. Mechanical Testing

Mechanical tests were carried out on an INSTRON 5965 universal testing machine (Illinois Tool Works Inc., Glenview, IL, USA) with a constant deformation speed of 1 mm/min at room temperature. Mechanical grips with notches were used to prevent slippage during the tensile tests. The number of independent elastic constants required for the complete determination of the compliance or stiffness matrix for an orthotropic material in the case of a plane stress state is four: *E*_1_, *E*_2_, *ν*_12_, and *G*_12_. According to ISO 527 [36], *E*_1_ and *E*_2_ can be determined from testing samples with raster angles of 0° and 90°, respectively. The shear modulus, *G*_12_, can be determined in tests with *θ* = 45°. In the case of uniaxial tension and taking into account (7),

(16) $$E_{x}(\theta )=\frac{\sigma_{x}}{\varepsilon_{x}}=\frac{1}{S_{11}^{*}}={\left[\frac{\cos^{4}\theta }{E_{1}}+\frac{\sin^{4}\theta }{E_{2}}+\frac{1}{4}\left(\frac{1}{G_{12}}-\frac{2\nu_{12}}{E_{1}}\right)\sin^{2}2\theta \right]}^{-1}$$

Then, *G*_12_ can be expressed from (9):

(17) $$G_{12}={\left[\frac{4}{E_{x}^{45^{{}^{\circ}}}}-\frac{1}{E_{1}}-\frac{1}{E_{2}}+\frac{2\nu_{12}}{E_{1}}\right]}^{-1}$$

where *E*^45^*_x_* is known from tests of samples with *θ* = 45°.

Poisson’s ratio, ν_12_, was determined during the tensile tests using the digital image correlation (DIC) method. A speckle (black matte enamel paint) was stochastically applied to the working part of the test sample with a raster angle of *θ* = 0° Since the samples were white-colored, the images were shot with a black-and-white filter to increase the contrast of the applied speckle. The sample was photographed for a range of elastic strains with a constant time step of 0.5 s. Then, the array of photos was uploaded to GOM Correlate 2022 software, where the quality of the surface with the applied raster on the working part of the sample was analyzed. The coordinate axes were oriented in such a way as to avoid distorting the results from the non-perpendicular installation of the camera in relation to the front surface of the sample. Next, the longitudinal (*ε_x_*) and transversal (*ε_y_*) strains in the working plane were calculated, and Poisson’s ratio was determined as follows:

(18) $$\nu =\left|\frac{\varepsilon_{y}}{\varepsilon_{x}}\right|$$

Since the elastic constants are related, a similar experiment was carried out for samples with *θ* = 0° to determine ν_21_. After that, the experimental and theoretical values of Poisson’s ratios were compared with each other.

In accordance with ISO 527 [36], the ultimate tensile strengths, *X* and *Y*, were determined from testing samples with raster angles of 0° and 90°, respectively. Two different methods were used to determine the shear strength, *W*. The first one is based on the Tsai–Hill criterion, and results were obtained for *θ* = 45°. In this case, *W*_1_ can be expressed as follows:

(19) $$W_{1}={\left[\frac{4}{{\left(\sigma_{x}^{max}\right)}^{2}}-\frac{1}{Y^{2}}\right]}^{-\frac{1}{2}}$$

The second one is based on ASTM D-3518 [37]:

(20) $$W_{2}=\frac{F_{\theta =45^{{}^{\circ}}}}{2S}$$

where *F*_*θ*=45°_ is the maximum load value when testing the sample with *θ* = 0° and *S* is the cross-sectional area of the sample.

## 3. Results

**Figure 3:**
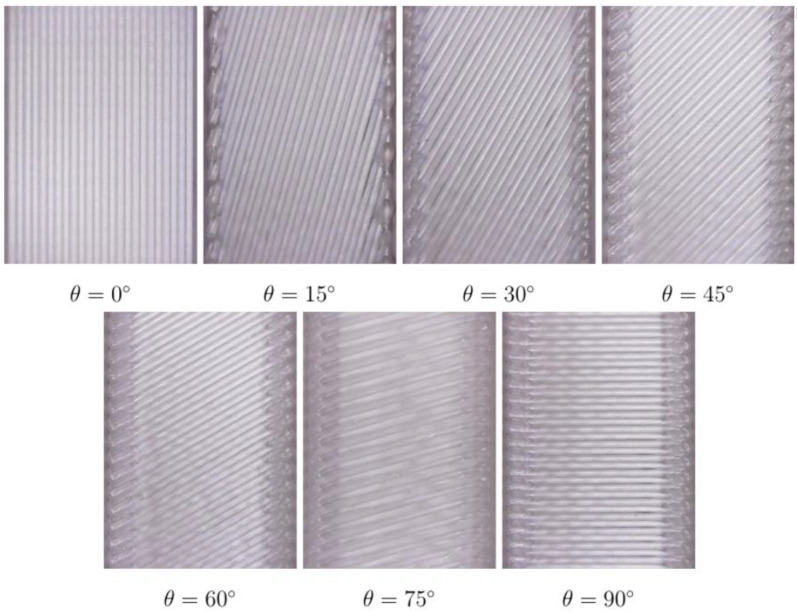
Photographs of the specimens with various raster angles.

The 3D-printed specimens are shown in Figure 3.

**Figure 4:**
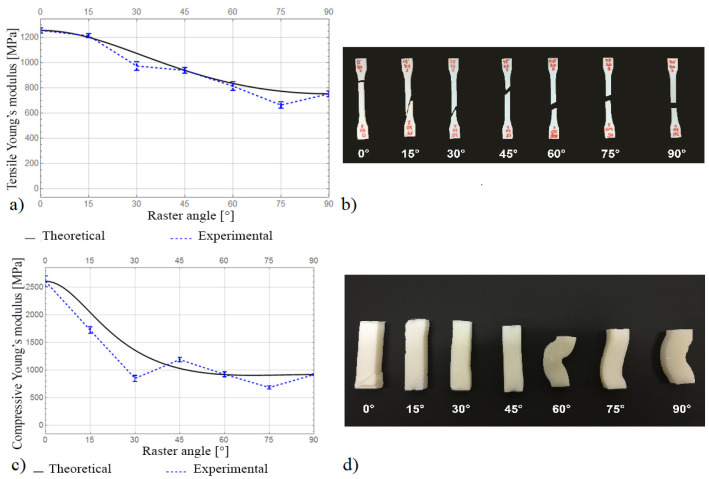
(**a**) Tensile Young’s modulus; (**b**) specimens after tensile tests; (**c**) compressive Young’s modulus; and (**d**) specimens after compression tests.

It can be seen that the selected printing parameters provide good in-plane adhesion. The burrs on the edges due to the nozzle deceleration are small. Figure 4 illustrates the experimental and theoretical results of the Young’s modulus evaluation in the tensile and compression tests.

It can be seen that the theoretical model predicts the values of the tensile Young’s modulus well over the entire range of raster angles. The higher theoretical values for samples with *θ* = 30° and *θ* = 75° could be caused by stress concentrations near the terminals. The relative errors in these cases are less than 15%. The experimental results of determining the compressive Young’s modulus correlate less with the theoretical estimate. This may be due to the presence of friction between the plates and the sample, as well as the buckling and kink during the test. The maximum relative error for the sample with *θ* = 30° was 31%.

**Figure 5:**
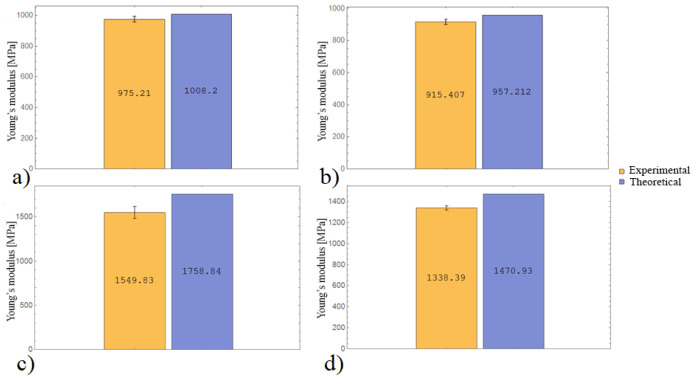
(**a**) Tensile Young’s modulus for *θ* = [0°, 90°, …]; (**b**) tensile Young’s modulus for *θ* = [0°, 45°, 60°, 90°, 0°, −45°, −60°, −90°, …]; (**c**) compressive Young’s modulus for *θ* = [0°, 90°, …]; and (**d**) compressive Young’s modulus for *θ* = [0°, 45°, 60°, 90°, 0°, −45°, −60°, −90°, …].

Figure 5 shows the results of the elastic modulus calculation for composite laminates with various raster angle according to the CLT.

The results of the theoretical and experimental calculations perfectly correlate with each other. In the tensile tests, the errors for the laminates were 3.3% (Figure 5a) and 4.4% (Figure 5b), respectively. In the compressive tests, the errors for laminates were 11.9% (Figure 5c) and 9% (Figure 5d), respectively. Thus, the CLT allows the stiffness values of samples under tension and compression to be predicted with good engineering accuracy.

**Figure 6:**
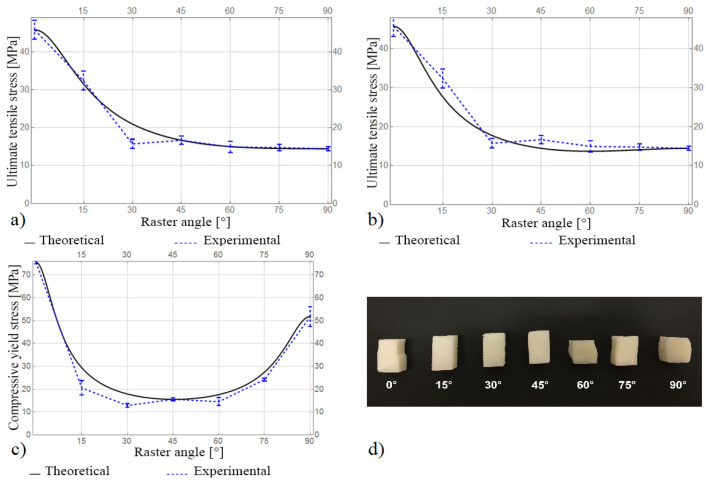
(**a**) Ultimate tensile strength calculated via *W*_1_; (**b**) ultimate tensile strength calculated via *W*_2_; (**c**) compressive yield stress; and (**d**) specimens after compression tests.

The experimental and theoretical results of the ultimate tensile strength and compressive yield stress using the Tsai–Hill criterion are shown in Figure 6.

The results using the first calculation method for the shear strength, *W*, predict the ultimate tensile strength of the samples with all values of the raster angle, except *θ* = 30° (Figure 6a). Since the fracture of these samples occurs near the grips, it causes a stress concentration and, as a result, understated value of the ultimate tensile stress. The results obtained using the second method for the shear strength, *W*, are also in good agreement with the experimental ones (Figure 6b). Nevertheless, these results differ significantly from the experimental results, so the first method, according to (19), was used to estimate the compressive yield stress. The implementation of the Tsai–Hill criterion makes it possible to predict the values of the compressive yield stress well (Figure 6c). As can be seen from the results, the Tsai–Hill criterion gives an overestimation of the compressive yield stress, which may be due to the buckling and kinking of the samples when the yield is reached (Figure 6d).

The Poisson’s ratios, *ν*_12_ and *ν*_21_, determined via DIC were 0.323 and 0.214, respectively. Taking into account the relation between the elastic moduli, *E*_1_, *E*_2_, and the Poisson’s ratios, *ν*_12_ and *ν*_21_ [27,33], *ν*_21_ = 0.194. In this case, the relative error was 10%, which is a satisfactory result.

## 4. Discussion

Despite all the limitations of the CLT, the results obtained in this research estimating the Young’s moduli of both the individual lamina and the composite laminate correlate well with experimental results. Setting up and carrying out experiments on uniaxial tension is much simpler than experiments on uniaxial compression. If, in the first case, the material is oriented in the direction of the applied load, then in the second, it tries to get out from under it [27]. Taking into account friction and buckling/kink effects during compression, the discrepancy between the theoretical approach and the experimental results is understandable. The obtained results are qualitatively and quantitatively correlated with similar results for the materials manufactured via FDM/FFF [23,24,25]. The motivation to study and theoretically describe the behavior of laminate composites not only under tension but also under compression is due to a significant difference in the values of the elastic modulus. When bending such composite beams, the neutral undeformed line shifts along the beam section, which makes the standard assessment of both the flexural modulus according to ASTM D7264 [38] and maximum stresses inapplicable. In this case, it is necessary to determine the position of the neutral line, which depends on the values of the tensile and compressive Young’s moduli. In the present research, the values of these moduli were *E*_1_ = 1255 MPa and *E*_2_ = 753 MPa under tension and *E*_1_ = 2609 MPa and *E*_2_ = 920 MPa under compression, respectively. That is, the ratio of moduli can be more than two times, which requires consideration when solving more complex bending problems that are not the subject of consideration in this paper.

The obtained values of Poisson’s ratio by the DIC method are in good agreement with the results of previous studies [39,40]. The Tsai–Hill criterion allows both the ultimate tensile strength and the compressive yield stress for materials manufactured using FDM/FFF 3D printing to be well predicted, despite the peculiarities of the behavior of samples during compression. It should be noted, the largest deviations were obtained for samples with a raster angle *θ* = 30°. In the case of the tensile tests, as shown above, this can be caused by the presence of a stress concentrator near the grips. In [27], it is demonstrated that during compression, the formation of a kink at an angle of 30 degrees is most energetically favorable, which may further explain the results obtained in the compression tests. In this case, it can be seen that the compressive yield stress has the smallest value in the range of raster angles.

Despite this, the application of the CLT and the Tsai–Hill criterion looks promising in the design and calculation of PLA parts manufactured via FDM/FFF 3D printing, both in tension and compression.

## Figures and Tables

**Table 1:** The 3D printing parameters.

Parameter	Value
Nozzle temperature, °C	215
Printing bed temperature, °C	60
Infill density, %	100
Printing speed, mm/s	40
Layer height, mm	0.25

## Data Availability

The data that support the findings of this study are available from the corresponding authors upon request.
